# Direct evidence for an expanded circulation area of the recently identified Balkan virus (Sandfly fever Naples virus species) in several countries of the Balkan archipelago

**DOI:** 10.1186/s13071-017-2334-y

**Published:** 2017-08-29

**Authors:** Nazli Ayhan, Bulent Alten, Vladimir Ivovic, Vit Dvořák, Franjo Martinkovic, Jasmin Omeragic, Jovana Stefanovska, Dusan Petric, Slavica Vaselek, Devrim Baymak, Ozge E. Kasap, Petr Volf, Remi N. Charrel

**Affiliations:** 1 UMR “Emergence des Pathologies Viralesˮ (EPV: Aix-Marseille Univ – IRD 190 – Inserm 1207 – EHESP – IHU Méditerranée Infection), Marseille, France; 2 Faculty of Science, Department of Biology, Ecology Division, VERG Labs, Hacettepe University, Beytepe, Ankara, Turkey; 3 University of Primorska, FAMNIT, Koper, Slovenia; 4 Faculty of Science, Department of Parasitology, Charles University, Prague, Czech Republic; 5 Faculty of Veterinary Medicine, Department of Parasitology and Parasitic Diseases with Clinics, University of Zagreb, Zagreb, Croatia; 6 Department of Parasitology, Veterinary Faculty of Sarajevo, Zmaja od Bosne 90, 71000 Sarajevo, Bosnia and Herzegovina; 7 Department of Parasitology and Parasitic Diseases, Faculty of Veterinary Medicine, Ss. Cyril and Methodius University, Skopje, Republic of Macedonia; 8 Faculty of Agriculture, Laboratory for Medical and Veterinary Entomology, University of Novi Sad, Novi Sad, Serbia; 9 National Institute of Public Health, Pristina, Kosovo

**Keywords:** Bunyaviridae, Phlebovirus, Arbovirus, Toscana virus, Meningitis, Fever, Sand fly, *Phlebotomus*, Phylogeny, Emergence

## Abstract

**Background:**

Recently, Balkan virus (BALKV, family *Phenuiviridae*, genus *Phlebovirus*) was discovered in sand flies collected in Albania and genetically characterised as a member of the Sandfly fever Naples species complex. To gain knowledge concerning the geographical area where exposure to BALKV exists, entomological surveys were conducted in 2014 and 2015, in Croatia, Bosnia and Herzegovina (BH), Kosovo, Republic of Macedonia and Serbia.

**Results:**

A total of 2830 sand flies were trapped during 2014 and 2015 campaigns, and organised as 263 pools. BALKV RNA was detected in four pools from Croatia and in one pool from BH. Phylogenetic relationships were examined using sequences in the S and L RNA segments. Study of the diversity between BALKV sequences from Albania, Croatia and BH showed that Albanian sequences were the most divergent (9–11% [NP]) from the others and that Croatian and BH sequences were grouped (0.9–5.4% [NP]; 0.7–5% [L]). The sand fly infection rate of BALKV was 0.26% in BH and 0.27% in Croatia. Identification of the species content of pools using *cox*1 and *cytb* partial regions showed that the five BALKV positive pools contained *Phlebotomus neglectus* DNA; in four pools, *P neglectus* was the unique species, whereas *P. tobbi* DNA was also detected in one pool.

**Conclusions:**

We report here (i) the first direct evidence that the Balkan virus initially described in coastal Albania has a much wider dissemination area than originally believed, (ii) two real-time RT-PCR assays that may be useful for further screening of patients presenting with fever of unknown origin that may be caused by Balkan virus infection, (iii) entomological results suggesting that Balkan virus is likely transmitted by *Phlebotomus neglectus*, and possibly other sand fly species of the subgenus *Larroussius*. So far, BALKV has been detected only in sand flies. Whether BALKV can cause disease in humans is unknown and remains to be investigated.

## Background

Phleboviruses (family *Phenuiviridae*) are arthropod-borne viruses transmitted by mosquitoes, ticks and sand flies to vertebrate hosts [1]. Several phleboviruses belong to the Sandfly fever Naples species complex (which include at least two human pathogens, namely Toscana virus causing neurological infections and Naples virus causing incapacitating febrile illness) [2]. In the Old World, sand fly-borne phleboviruses are transmitted by *Phlebotomus* spp*.* and *Sergentomyia* spp*.* and show a wide distribution in all countries of the Mediterranean basin [2], http://ecdc.europa.eu/en/healthtopics/vectors/vector-maps/Pages/VBORNET_maps_sandflies.aspx. During the last decade, several new phleboviruses were discovered in Mediterranean countries either in sand flies [3–9] or clinical samples [10]. Each was genetically related to any of the three following groups (based on antigenic relationships): Sandfly fever Naples species, Salehabad and Sandfly fever Sicilian/ Corfou virus group. In the Balkans, the current knowledge on circulating phleboviruses is limited. Recently, the Balkan virus (BALKV) was discovered in sand flies collected in Albania and genetically characterised as a member of the Sandfly fever Naples species complex [11]. Two specific quantitative real-time RT-PCR assays were designed to screen entomological specimens collected in the surrounding countries, i.e. Croatia, Bosnia and Herzegovina (BH), Kosovo, Republic of Macedonia (RoM), and Serbia, to gain knowledge concerning the geographical area where exposure to BALKV exists.

## Methods

Sand flies were collected in the field in 2014; 10 stations in Kosovo and 8 stations in Serbia, in 2015; 5 stations in Croatia, 6 stations in BH, 5 stations in RoM, 1 station in Montenegro and 1 station in Serbia (Table 1) using a previously described method [11]. Traps were placed near animals with the consent of the owners. BALKV RNA was detected using 2 SYBR Green real-time RT-PCR specific assays targeting the polymerase gene (BALKV-L-F; 5′-CTD ATY AGY TGC TGC TAC AAT G-3′, BALKV-L-R; 5′-CCA TAA CCA AGA TAY TCA T-3′) and the nucleoprotein gene (BALKV-S-F; 5′-AGA GTR TCT GCA GCC TTT GTT CC-3′, BALKV-S-R; 5′-CAG CTA TCT CAT TAG GYT GT-3′). The cycling program consisted of 50 °C for 30 min and 95 °C for 15 min, followed by 40 cycles at 94 °C for 15 s, 60 °C for 30 s, and 72 °C for 45 s, with a final melting curve step at 95 °C for 1 min, 60 °C 30 s and 95 °C for 30 s. Melting curves for positives were at 75 °C for the polymerase assay and 79.5 °C for the nucleoprotein test.

**Table 1:** Trapping campaigns and geographical information of the Balkan virus positive pools

Trapping region	Number of collected sand flies			No. of pools (positive pools_gender)
	Female	Male	Mix/unknown	
2014: Kosovo				
Vermice	12	16	4	6
Zhur	10	4	2	6
Landrovice	11	14	2	6
Krusha E. Vogel	0	0	2	1
Junik	28	27	3	7
Donji Livoc	1	0	0	1
Cernice	2	0	0	1
Nishor	2	4	0	2
Studencan	18	11	2	6
Semetiste	50	43	2	12
Total	134	119	17	48
2014: Serbia				
Aleksinac / Kraljevo	8	8	0	2
Brest	3	3	0	2
Arbanasce	8	0	0	1
Prugovac	5	5	0	2
Subotinac	0	1	1	2
Mozgovo	0	1	0	1
Bovan	2	0	0	1
Jugbogdanovac	8	0	0	2
Total	34	18	1	13
2015: Bosnia and Herzegovina				
Sovici	105	77	0	11//(#B1_male)
Mikanjici	22	18	0	4
Zakovo	10	3	0	2
Grab	47	0	0	5
Stolac	55	44	0	7
Tuli	0	5	0	1
Total	239	147	0	19
2015: Croatia				
Duba	176	129	30	18/(#C13_male)
Jesenice	81	0	25	6
Gorna Ljuta	22	18	2	4
Zvekovica	12	9	0	3
Vidonje	490	55	404	47/(#C41, #C50, #C51_females)
Total	781	211	461	78
2015: Montenegro				
Ozrinici	21	14	2	4
Total	21	14	2	4
2015 Romania				
Mokrino	48	91	3	26
Kezhovica	85	10	42	15
Dedeli	25	7	1	10
Suvo Grlo	274	0	0	30
Furka	11	4	1	4
Total	443	112	47	85
2015: Serbia				
Krasava	19	9	1	16
Total	19	9	1	16
Grand total	1671	630	529	263

Phylogenetic relationships were reconstructed using sequences of the S and L RNA segments. Positive samples were PCR-amplified targeting a portion of the polymerase [12] and the nucleoprotein genes [13, 14] (two systems producing overlapping sequences which were concatenated before analysis). Sand fly species identification within positive pools was performed using as previously described cytochrome *c* oxidase subunit 1 (*cox*1) and cytochrome *b* (*cytb*) barcoding gene regions followed by NGS sequencing of the corresponding PCR products [11]. A 50 μl-volume of BALKV positive pools was inoculated onto Vero cells for attempting virus isolation [7, 9].

## Results

**Fig. 1:**
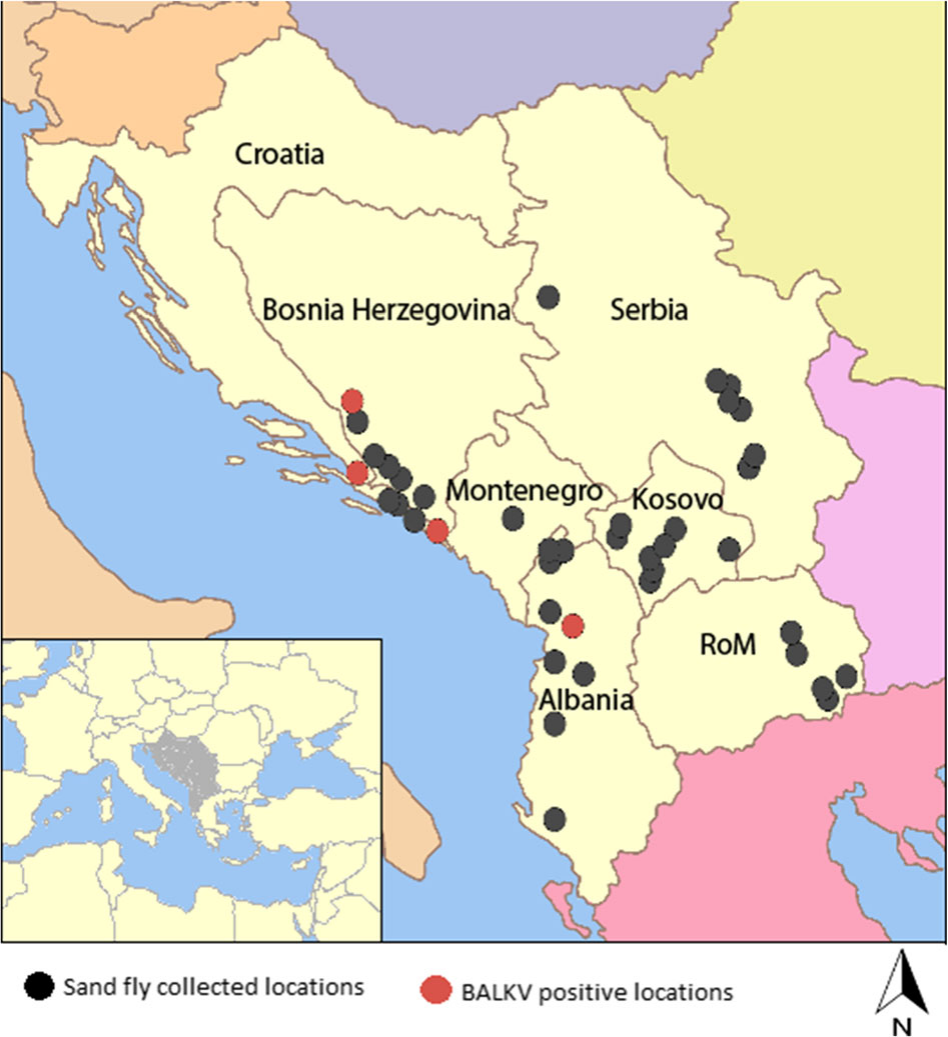
Geographical distribution of the sand fly trapping stations. Black circles denote trapping stations. Red circles denote trapping stations in which at least one pool was found to contain Balkan virus RNA

In 2014 a total of 270 and 53 sand flies were collected from Kosovo and Serbia, respectively. In 2015, 1453, 386, 37, 602 and 29 sand flies were trapped in Croatia, BH, Montenegro, RoM and Serbia, respectively (Table 1). BALKV RNA was detected in 4 pools from Croatia (3 collected in Vidonje [C41, C50, C51 at 42.98244N, 17.64294E (240 m)], 1 in Duba [C13 at 42.60032N, 18.33946E (475 m)]) and in 1 pool from BH in Sovici (B1 at 43.408240N, 17.329175E, 283 m) (Table 1, Fig. 1).

**Fig. 2:**
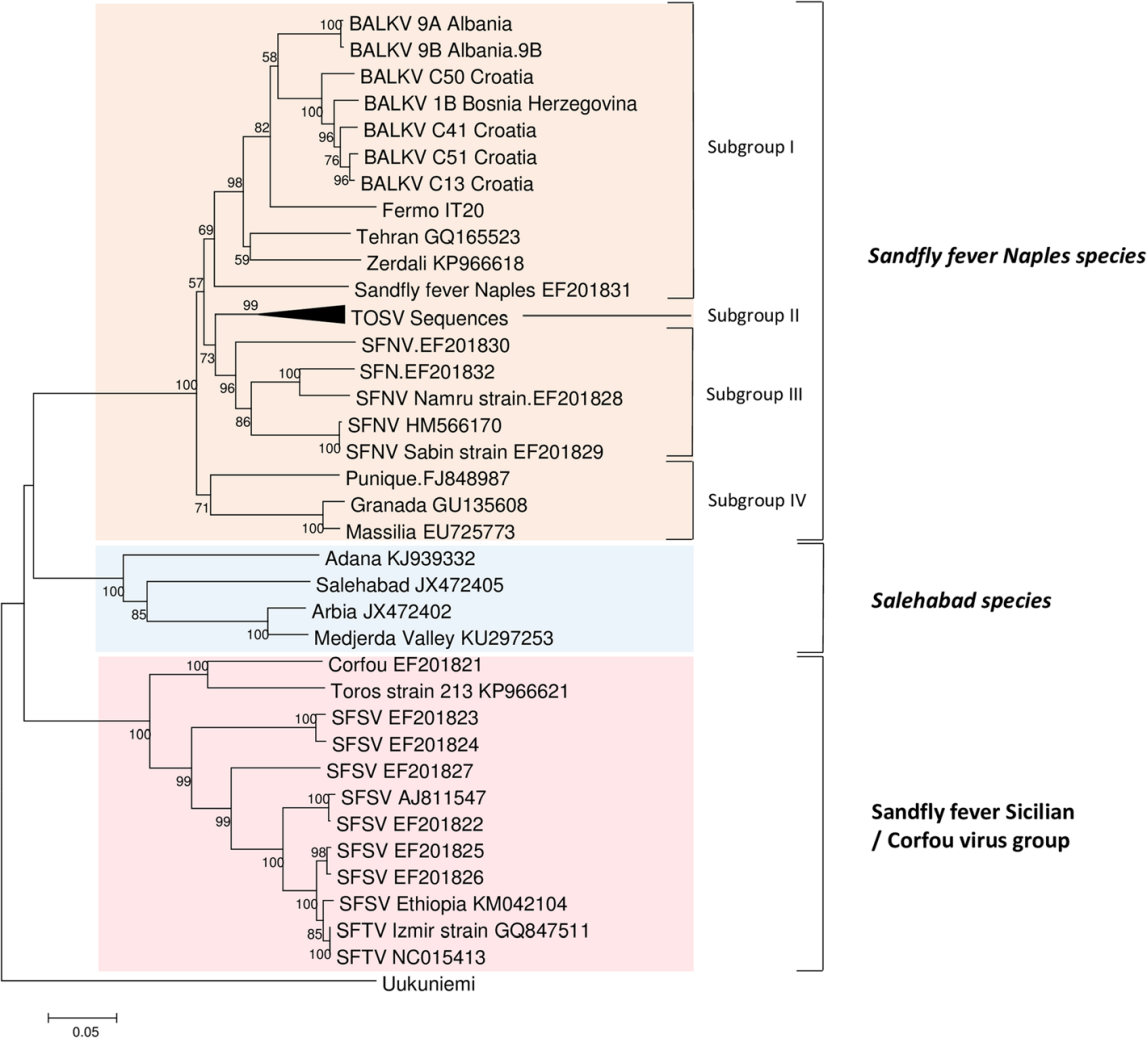
Phylogeny of the Balkan virus and closely related phleboviruses using partial nucleotide sequences of the nucleoprotein gene (572 nt). Neighbor-joining analysis (Kimura 2-parameter model) was performed using MEGA6, with 1000 bootstrap replicates

**Fig. 3:**
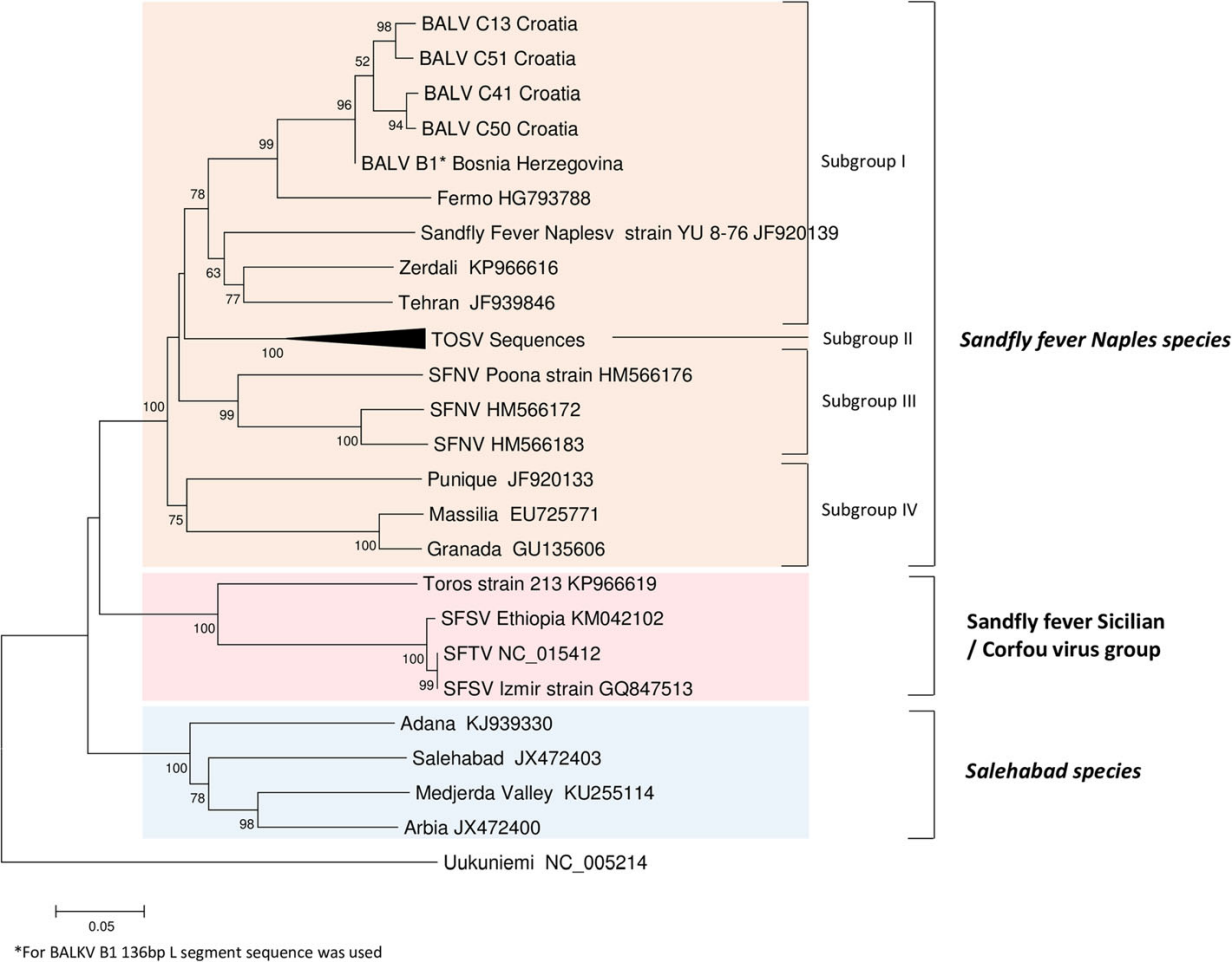
Phylogeny of the Balkan virus and closely related phleboviruses using partial nucleotide sequences of the polymerase gene (525 nt). Neighbor-joining analysis (Kimura 2-parameter model) was performed using MEGA6, with 1000 bootstrap replicates

Although not quantitative, the low C_t_ values observed with the polymerase gene (C_t_ range 19.9–24.4) and the nucleoprotein gene (C_t_ range 19.8–32.8) SYBR Green real-time RT-PCR was indicative of high viral load in the positive pools. Phylogeny was reconstructed by using sequences in the S and L RNA segments that were 572 nt (Fig. 2) and 525 nt long, respectively (Fig. 3). Identical groupings were observed using both markers. BALKV formed a homogenous cluster with common ancestor supported by a high bootstrap value. BALKV was included in the subgroup I of the Sandfly fever Naples species complex together with SFNV, Tehran, Zerdali and Fermo viruses.

For pool B1, failure to obtain a positive PCR with Nphlebo primers led us to sequence the 136 bp SYBR Green RT-qPCR product for genetic and phylogenetic analysis. Study of the diversity between BALKV sequences from Albania, Croatia and BH showed that (i) Albanian sequences were the most divergent (9–11% [NP]) from the others, and (ii) that Croatian and BH sequences were grouped (0.9–5.4% [NP]; 0.7–5% [L]) (GenBank: KY662276–KY662287).

Identification of the sand fly species contained in the BALKV-positive pools detected *Phlebotomus neglectus* sequences in all five pools; *P. neglectus* was the unique species in four pools, whereas *P. tobbi* DNA was present in 1 pool from Croatia (Table 2).

**Table 2:** Details of the Balkan virus positive pools with sandfly species identification using cytochrome b and cox1 sequences

Trapping locality	Pool code	Sand fly species	Gene	Reads	No. of sand flies	Gender	Collection date	Altitude (m)
Bosnia and Herzegovina								
Sovici	B1	*P. neglectus*	*cytb*	1427	27	male	06/07/2015	283
			*cox*1	4257				
Croatia								
Duba	C13	*P. tobbi*	*cytb*	1211	20	male	13/07/2015	475
			*cox*1	546				
		*P. neglectus*	*cytb*	967				
			*cox*1	7351				
Vidonje	C41	*P. neglectus*	*cytb*	950	20	female	16/07/2015	240
			*cox*1	8182				
Vidonje	C50	*P. neglectus*	*cytb*	1834	20	female (bf)	16/07/2015	240
			*cox*1	5302				
Vidonje	C51	*P. neglectus*	*cytb*	3143	20	female (bf)	16/07/2015	240
			*cox*1	22,867				

## Discussion

The Balkan Peninsula is the region where sand fly fever was first described at the end of the nineteenth century in BH [15, 16]. Subsequent studies provided direct and indirect evidence for the presence of viruses belonging to the SFNV in BH [17–21]. In Croatia, antibodies against SFNV were found in human populations, with highest rates (up to 53.9%) observed on islands and in coastal regions [18, 22–27]. BALKV belongs to the Sandfly fever Naples species complex where it is most closely to Fermo, SFNV YU 8–76, Zerdali and Tehran viruses isolated in Italy, Serbia, Turkey and Iran which are grouped in the subgroup I [6, 9, 19, 28] (Figs. 2, 3). BALKV was first detected from two sand fly pools from Albania, Kruje region [11]. Here, we demonstrated that BALKV has a much larger circulation area that seems to be confined to the Adriatic coast of the Balkan Peninsula. This merits further confirmation through similar studies conducted north and south of the current study area (Fig. 1).

To our knowledge, BALKV is the first phlebovirus to be genetically identified in BH. Assuming that each positive pool contained one infected sand fly only, the sand fly infection rate of BALKV is 0.26% in BH and 0.27% in Croatia; which is higher than Zerdali virus (0.035%) and similar to Fermo virus (0.20%) [6, 9]. Identification of the species content of pools using *cox*1 and *cytb* showed that *P. neglectus* is the only species to be found in all BALKV RNA positive pools; indicating that this species might be the vector of BALKV. Interestingly, *P. neglectus* belongs to subgenus *Larroussius*, similar to *P. tobbi* which seems to be a typical vector for Zerdali virus, another member of the *Sandfly fever Naples species* [9]. Together, these data support the hypothesis that *Larroussius* sand flies are typical vectors of the members of this virus group.

## Conclusions

We report here (i) the first direct evidence that Balkan virus initially described in Coastal Albania has a much wider dissemination area than originally believed, (ii) two real-time RT-PCR assays that may be useful for further screening of patients presenting with fever of unknown origin that may be caused by Balkan virus infection, (iii) entomologic results suggesting that Balkan virus is likely transmitted by *Phlebotomus neglectus*, and possibly other sand fly species of the subgenus *Larroussius*. So far, BALKV has been detected only in sand flies. Whether BALKV can cause disease in humans is unknown and remains to be investigated.

## Abbreviations

BALKV: Balkan virus
BH: Bosnia and Herzegovina
L: Large RNA segment
NGS: Next generation sequencing
NP: Nucleoprotein
RoM: Republic of Macedonia
RT-PCR: Reverse transcriptase polymerase chain reaction
S: Small RNA segment
